# Survival Outcomes and Care Equity among Patients with Advanced Breast Cancer in Auckland, New Zealand

**DOI:** 10.1155/2022/7116040

**Published:** 2022-11-07

**Authors:** Edmond Ang, Dug Yeo Han, Sheridan Wilson

**Affiliations:** ^1^Te Pūriri o Te Ora Auckland Regional Cancer and Blood Service, Auckland District Health Board, New Zealand; ^2^Starship Hospital, Auckland District Health Board, New Zealand

## Abstract

**Aim:**

The Auckland Advanced Breast Cancer Review (AABC) was a review of patients diagnosed with advanced inoperable/metastatic breast cancer (ABC) within the Auckland region of New Zealand, commissioned in response to a Breast Cancer Registry report (BCFNZR) that showed poor and inequitable survival outcomes. The review was aimed at assessing equity of care and identifying healthcare delivery gaps for patients with ABC in the Auckland region.

**Method:**

In this retrospective study, patients living within the Auckland region, diagnosed with ABC between the 1st January 2013 to the 31st December 2015 were identified from the Breast Cancer Registry. Data censorship date was 30th January 2019 to allow a minimum of 3 years of follow-up. Demographic, diagnostic, treatment, and survival data were extracted from electronic records for statistical analysis.

**Results:**

Of the 388 patients that met inclusion criteria for this study, median overall survival (medOS) was 18.9 months in the total population, with no difference between patients with de novo metastatic disease (dnMBC -18.9 m) and recurrent metastatic disease (rMBC -18.7 m). No statistically significant differences in medOS was found amongst Maori (16.2 m), Pacific People (17.3 m), and NZ European (18.9 m) or when patients were stratified according domicile district health board. Median number of lines of systemic treatment was two, with similar treatment exposure between ethnic groups.

**Conclusion:**

While treatment uptake and survival outcomes were generally comparable across ethnicity and district health boards, dnMBC survival outcomes were considerably poorer than expected, earmarking this subset of patients with ABC for more in-depth research.

## 1. Introduction

Approximately 300 patients are diagnosed with advanced breast cancer (ABC) in New Zealand (NZ) annually. Despite improvements in treatment options and survival outcomes in recent years, NZ ABC mortality rates continue to exceed the OECD average [1]. In a recent Global Burden of Disease report, breast cancer retained its position as one of the top ten leading causes of death in the country [2].

Paucity of data has curtailed efforts to improve ABC patient outcomes in NZ. To address this gaping issue, the Breast Cancer Foundation of NZ (BCFNZ) published *I Am Still Here (BCFNZR)* in September 2018, NZ's first comprehensive report on the state of ABC in the country [3]. The report was based on the output of three studies commissioned by the foundation. Central to the report was a survival analysis of ABC cases registered in the Breast Cancer Foundation National Registry (BCR) between the year 2000-2015.

Despite observable gains in survival outcomes over the 15-year period, the BCFNZR findings were disappointing. The composite median overall survival (medOS) of women in NZ with ABC was 16 months, while one-year and five-year survival rates were documented at 46% and 12%, respectively. These figures are considerably lower, when compared to contemporary studies published worldwide, with the gap between NZ and other similar jurisdictions widening over recent years [4, 5]. Interestingly, the metastasis free interval (mFI: 30 months) amongst patients who developed metastatic relapse after early breast cancer was similar to international standards. This highlights out ABC care as an area requiring prioritisation.

Improving health equity has been a central focus of policymakers in NZ over the last two decades. Unfortunately, inequitable health outcomes remain pervasive [6]. Maori health outcomes have historically lagged behind non-Maori across a range of cancer and noncancer related indicators [7]. This inequity was reflected in the BCFNZR, where Maori had the poorest survival outcomes amongst patients with ABC compared to all other ethnicities in NZ, with a medOS of only 12.8 months. Migration has also resulted in considerable changes to the nation's demographics. Consequently, there is considerable interest among health policymakers to ensure that all ethnic minorities, particularly Pacific Peoples receive equitable care.

Auckland is New Zealand's largest and most ethnically diverse city. The city accounts for a third of the nation's population, and houses the largest Polynesian population of any city in the world [8]. Publicly funded universal health coverage in the Auckland region is delivered by three separate district health boards (Auckland: ADHB, Waitemata: WDHB, and Counties Manukau: CMH). The ethnic composition of the population served by each district health board varies in tandem with the asymmetrical geographical distribution of ethnic groups within the city. For instance, CMDHB serves a much larger proportion of Pacific Peoples. For patients with ABC, initial surgical care is provided by their respective district health boards based on their residential address. On the contrary, special medical and radiation oncology care was provided by a central regional service based at ADHB. The complex integration of cancer care provision across these distinct health boards created unique challenges in the provision of equitable care geographically.

The Auckland Advanced Breast Cancer Review (AABC) is a retrospective review of patients diagnosed with ABC within the Auckland region over a three-year period. The study was commissioned by the Te Pūriri o Te Ora Auckland Regional Cancer and Blood Service in response to the BCFNZR. The objectives of the review was threefold: (i) to externally validate the accuracy of the outcomes reported in the BCFNZR for the Auckland region, (ii) to determine if equitable care was provided across ethnicities and DHBs, and (iii) to identify potential healthcare delivery gaps that may direct further research and resource allocation.

## 2. Methodology

The BCR has been accruing data from 5 regions in New Zealand, including the Auckland region, since its establishment in 2000. This comprehensive electronic database includes demographic, diagnostic, treatment, and survival data from both public and private sector patients. The registry was the source of data used in the BCFNZR.

With the approval of our local institutional ethics board, we extracted the unique National Health Index of patients with advanced, inoperable, and/or metastatic breast cancer living within the Auckland region who were diagnosed between the 1st January 2013 to the 31st December 2015 and who were included in the BCR.

A comprehensive electronic chart review was conducted. Demographic, diagnostic, treatment, and survival data were extracted. Investigators of the AABC review used an agreed data entry guideline to define and support uniformity of data collection. The date of data censorship was set on the 30th January 2019 to allow a minimum of 3 years of follow-up from ABC diagnosis. Patients who were diagnosed outside of the study period, had insufficient information recorded in their electronic data or had moved out of the Auckland region were excluded.

Date of ABC diagnosis was the date the investigator deemed a conclusive ABC diagnosis was made either by clinical findings, tissue sampling, or radiological investigations. All patients who had confirmed metastatic disease within three months of an early breast cancer were categorised as having de novo metastatic disease (dnMBC).

Descriptive statistics were used to summarise the cohort and time to event analysis was presented by Kaplan-Meier survival curves. Statistical analyses were carried out using SAS 9.4 (SAS Institute Inc., Cary, NC, USA) and R (R Core Team (2021). R: A language and environment for statistical computing. R Foundation for Statistical Computing, Vienna, Austria. URL https://www.R-project.org/).

## 3. Results

A total of 517 cases were extracted from the database of which 388 cases met inclusion criteria and 129 were excluded. Of 129 excluded cases: 97 were outside of study periods, 12 had inadequate information, 17 were outside of region, and three did not have breast cancer.

Demographic and clinical characteristics of our cohort are presented in Table 1. The number of new ABC cases per year over the 3-year period was similar. Age at time of ABC diagnosis ranged from 28 to 97 years with a median of 60 years. The ethnic composition of ABC cases broadly mirrored the ethnic composition of Auckland (NZ European: 60%, Maori: 9%) with an excess of ABC in Pacific People (17%) and a substantially smaller percentage of Asians (13%). The proportion of patients by DHB were similar (ADHB: 29%, WDHB: 35%, and CMDHB: 36%). Thirty percent of ABC cases were dnMBC presentations as opposed to 22.7% in the BCFNZR. The metastasis free interval was 40 months (BCFNZR: 30 months) in patients with relapsed metastatic breast cancer (rMBC). Case distribution by disease subtypes were consistent with expectations and are shown in Table 1.

**Figure 1:**
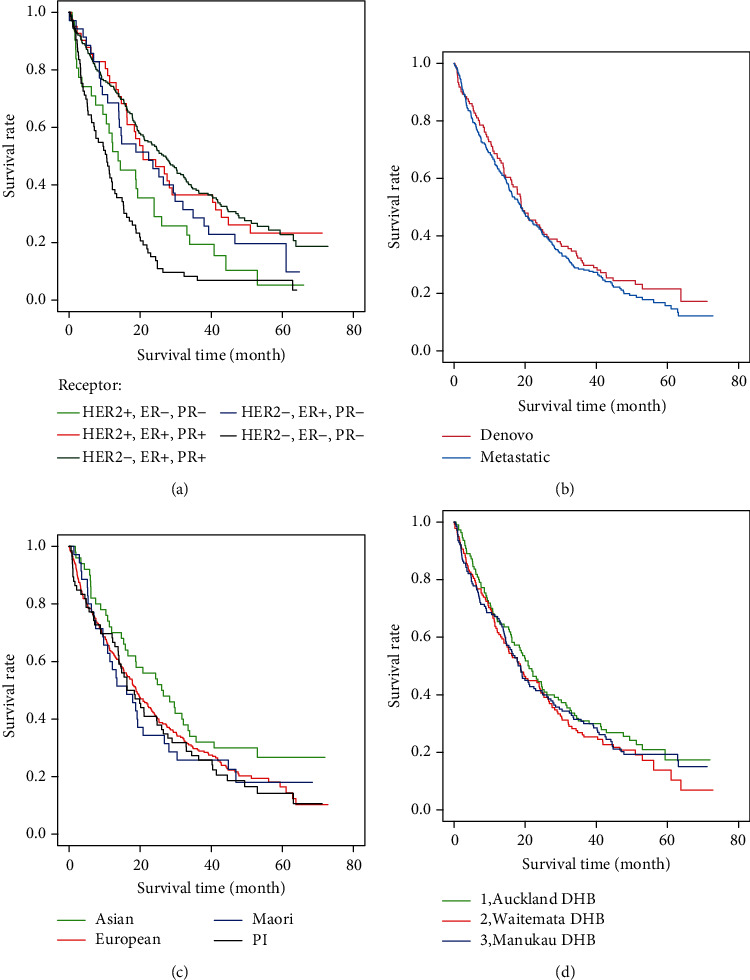
Survival curves based on (a) receptor subtypes, (b) De novo versus relapsed metastatic, (c) ethnicity, and (d) domiciliary district health board.

Survival data are summarised in Table 2 and Figure 1. At the date of data censorship, 77 of the 388 cases were alive. The medOS was 18.9 months, with one year survival and three-year survival at 66% and 29.4%, respectively. Survival outcomes were surprisingly similar in the dnMBC (medOS: 18.9 m, one year survival: 65%, and three-year survival: 31.4%) and rMBC (medOS: 18.7 m, one year survival: 65%, and three-year survival: 28%) group. Survival by receptor subgroups was as expected with the most favourable survival outcome documented in the hormone positive, HER2 negative subgroup (25.3 m). Survival outcomes for the triple negative subgroup were least favourable (10.6 m). There were no statistically significant differences in medOS amongst Maori (16.2 m), Pacific People (17.3 m), and NZ Europeans (18.9 m); Asian patients had the most favourable medOS (26.3 m).

**Figure 2:**
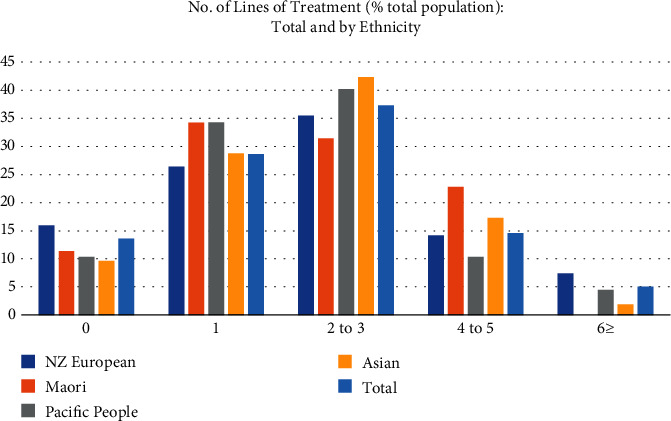
Number of lines of systemic treatment for ABC patients in Auckland 2013-2015.

Figure 2 summarises the number of lines of systemic treatment received by patients with ABC in between 2013-2015. The median number of lines of systemic treatment was 2. A larger proportion of Maori patients had at least one line of systemic treatment (89%) compared to that reported for the Auckland region in the BCFNZR (75%). Twenty-three percent of Maori patients received four or five lines of systemic treatment. Similar treatment exposure in terms of lines of therapy was documented for Pacific Peoples with 90% receiving at least a single line of systemic treatment. At censorship, 67 of the 77 patients that were still alive remained on treatment. Only thirteen percent of the patients did not receive any systemic treatment postdiagnosis, a considerably lower percentage when compared to that which was attributed to the Auckland region in the BCFNZ report (31%). Poor performance status (55%) and patient's personal decision (21%) were the commonest primary reasons cited for not receiving any systemic treatment. Poor performance status was also the commonest reasons cited for stopping systemic treatment after at least 1 line of therapy (44%).

The present study indicated less favourable outcomes for dnMBC than was expected. Logistic regression analysis was conducted to identify potential variables that could account for the discrepancy. De novo metastatic disease was associated with age (OR = 1.02, CI: 1.01-1.03, *p* = 0.0462), Asian ethnicity (OR = 2.54, CI: 1.31-4.91, *p* = 0.0416), and advanced metastatic disease at diagnosis (5 > metastatic sites at diagnosis: OR: 5.49. CI: 1.69-17.8). Thirty-three percent of patients were aged above 70, and 19% were aged above 80 in the dnMBC subgroup as opposed to 25% and 10.7% in the rMBC subgroup, respectively. The majority of patients aged 80 and above with dnMBC were symptomatic, had poor functional status and suffered from multiple comorbidities at presentation. The medOS of this group of patients was 9.4 months from diagnosis. De novo metastatic disease was associated with advanced disease at presentation, with 34% presenting with locally advanced disease involving the chest wall and 9% with extensive metastatic disease involving 5 or more organ sites compared to 12% and 2% in the rMBC subgroup, respectively. Receptor subtype and number of systemic treatment lines did not appear to differ between de novo and relapsed metastatic group.

## 4. Discussion

The Auckland Advanced Breast Cancer Review (AABC) is the first comprehensive review of ABC outcomes in the Auckland region since the publication of the NZBCFR. While the BCFNZR provided a global overview of ABC care and outcomes in NZ, our study provides a unique perspective that focuses on ABC patient experience in a largely urban and ethnolinguistically diverse population. Overall, the results from our study were largely consistent with the outcomes reported in the BCFNZR, externally validating its accuracy and valid. This study reinforces the pivotal role of the BCR, in the research and advancement of breast cancer care in NZ. The sobering results unfortunately confirm the inferior ABC survival outcomes in NZ as reported in the BCFNZR. The cause of this poor survival outcome is likely to be multifaceted.

There were a few noteworthy differences between the BCFNZR and our study. Firstly, treatment uptake rates attributed to the Auckland region were substantially lower in BCFNZR. We believe this reflects difficulty in accurately capturing receipt of therapy in the present BCR, particularly in the case of oral therapies for which there are a number in ABC. Secondly, in contrast to the BCFNZR, our study did not reveal a statistically significant difference in treatment uptake and survival outcome among Maori, Pacific People, and NZ Europeans. While this is reassuring, the number and proportion of Maori patients in our study was small (*n* = 35, 9%), reflecting the urban ethnic composition of Auckland. This is unlikely to represent the experience of Maori patients with ABC living in more rural communities nationwide.

Perhaps the most striking difference is the substantially larger percentage of dnMBC presentations and the unexpectedly poorer survival outcome of this population in our study compared to national data. Patients with dnMBC are expected to have better survival outcomes compared to rMBC attributed to treatment naivety at presentation [9, 10]. On the contrary, we report similar survival outcomes in both dnMBC and rMBC subgroups. We have confirmed that our definition of de novo metastatic disease is consistent with the definition used in the BCR and that registry data in fact confirms dnMBC rates to be 35% in the Auckland region.

De novo metastatic breast cancer is increasingly viewed as a distinct entity with a different tumour biology and disease course to rMBC, with overrepresentation of advanced and aggressive disease forms [11]. De novo metastatic disease has also been linked with unfavourable social determinants of health, such as delayed diagnosis, poor health literacy, and poor cancer screening [12, 13]. Rates of dnMBC diagnosis have been reported to range from 3-6% in high income countries to 10-30% in low and middle income countries with associations with advanced age, nonwhite race, and lower socioeconomic status [14].

Interesting socioeconomic and ethnic undertones appear to be present in the dnMBC subgroup in our study and awaits elucidation. While the proportion of Asian (40%) and Pacific Peoples (38%) were overrepresented in this category as compared to NZ Europeans (26%), Asian patients had survival outcomes (medOS: 26 m) that were closer to that which is expected in dnMBC (26 m), compared to Maori (medOS: 16 m) and Pacific Peoples (medOS: 15 m). The overall poorer survival outcomes amongst patients with dnMBC in this study may stem from the fact that a larger proportion of patients in the dnMBC subgroup were elderly, comorbid and possessed more advanced disease. In fact, almost a third of patients in this subgroup presented with clinically visible, mutilating chest wall disease, indicating delayed presentation.

There are limitations to this retrospective study. Firstly, patients with ABC were identified from the BCR, which is the same data source used in the BCFNZR, potentially missing patients who may have not been captured in the registry. Secondly, data extraction errors from the registry and the absence of complete electronic data particularly among patients who received treatment in private centres or those who migrate in and out of Auckland, may impact on data accuracy. Data on individual patient access to private cancer care, unfunded therapies, clinical trial participation, and the consequent impact this may have had on survival outcomes was not specifically collected or analysed. Next, quality of life is a crucial indicator of cancer care quality, particularly amongst patients with metastatic disease. Regrettably, patient reported outcomes (PROs) are not consistently captured in our service and therefore not reportable in this study. Lastly, this data is not necessarily reflective of current standards of care due to advances in systemic therapy options. For instance, the poor survival outcome reported amongst patients with hormone receptor negative, HER-2 positive subgroup is likely to have improved substantially with improved access to novel HER-2 directed treatment options.

ABC survival outcomes were similar across the three DHBs served by Te Pūriri o Te Ora despite demographic differences in these geographical regions. While this is encouraging, this does not necessarily reflect equity of care provision, particularly as we are unable to identify patients who have not accessed our health systems for their ABC. Moreover, most oncology consultation and treatments continue to be administered in Auckland City Hospital, which is located in central Auckland. This is expected to influence disparities in treatment access for those domiciled far from this centre. Qualitative studies and the use of PROs, assessing quality of life, time, and financial toxicity may be helpful to assess broader aspects of equity of care in the future.

## 5. Conclusion

Improving outcomes for patients with ABC in an equitable way and lifting outcomes across the entire strata of the ABC community regardless of urban-rural or ethnolinguistic position remains a formidable challenge. The establishment of a national breast cancer registry and the planned 5 yearly report on the state of ABC care and outcomes by the BCFNZ are pivotal steps for stimulating targeted research and spurring initiatives to improve care delivery and access to new therapeutic options. Local and regional studies such as the AABC are complimentary and can be useful to identify locally relevant challenges and inspire locally designed interventions. Our study has identified the dnMBC subgroup in Auckland as requiring further consideration and has highlighted the need for future research to understand the influences that shape stage at presentation and treatment decisions following a diagnosis of ABC.

## Figures and Tables

**Table 1:** Key demographic and disease features of patients diagnosed with ABC in Auckland 2013-2015.

Number of cases per year	2013	131
	2014	126
	2015	131

Age	Median: 60.6 years	60.6 years
	Range	28-97 years

Ethnicity	European	60%
	Māori	9%
	Asian	13%
	Pacific people	17%

Presentation	De novo (dnMBC)	30%
	Relapse metastatic (rMBC)	70%

Receptor subtypes	Hormone receptor +, HER2-ve	60%
	HER2+	21%
	Triple negative	19%

Lines of treatment	Median	2
	Patients who did not receive any systemic treatment	13.7%

**Table 2:** Key survival outcomes.

Parameter	Subgroups	*n*	medOS(LQ-UQ) in months
Relapsed versus De novo	rMBC	270	18.7 (7.0-39.1)
	dnMBC	118	18.9 (9.2-41.7)

Receptor subtypes	ER + PR + HER2-	202	26.9 (11.4-44.7)
	ER + PR-HER2-	35	22.4 (8.9-39.3)
	ER-PR- HER2+	31	13.8 (3.6-33.1)
	ER + PR + HER2+	33	20.8 (13.2-44.8)
	Triple negative	73	10.6 (3.9-18.7)

Ethnicity	European	232	18.9 (7.4-39.4)
	Māori	35	16.2 (7.0-38.9)
	Asian	50	26.3 (10.8-46.8)
	Pacific peoples	66	17.3 (6.9-39.0)

No. of lines of treatment	None	53	3.3 (1.6-9.6)
	1	111	9.7 (4.7-31.7)
	2-3	145	20.8 (14.4-41.2)
	4-5	57	32.4 (25.3-43.8)
	6+	21	47.6 (32-59.1)

## Data Availability

Electronic data used to support the findings of this study are restricted by the Auckland City Hospital Research Board in order to protect patient confidentiality.
